# In search of Pan-American indigenous health and harmony

**DOI:** 10.1186/s12992-019-0454-1

**Published:** 2019-02-20

**Authors:** Julie Babyar

**Affiliations:** Vallejo, USA

**Keywords:** Indigenous health, Indigenous rights, Latin American healthcare, North American health

## Abstract

The objective of this article is to describe the state of North, Central, South American and Caribbean (Pan-American) indigenous health. The second objective is to identify recommendations for optimal healthcare and research strategies to achieve indigenous health equity. Current health disparities continue to present between indigenous populations and general populations. Research foci of Pan-American indigenous health center on health outcomes for chronic and acute disease as well as presence of indigenous in data sets. Research is both qualitative and quantitative. Recommendations to improve indigenous health in effort of health equity are variable yet feasible. Stronger epidemiology, continued cohesive Pan-American global strategies, better research alignment with emphasis to quality and comprehensive metric analyses in healthcare delivery are all avenues to improve the health of the indigenous. Research and healthcare delivery on the Pan-American indigenous must be maximized for optimal results, must be representative of the indigenous communities, must be implemented in best practice and must introduce sustainable healthcare delivery for Pan-American indigenous health equity.

## Background

Indigenous people are commonly defined as those who are descendants of a geographic location or territory; those present before a new, dominant population arrived. Indigenous people retain social, economic, political and cultural characteristics of their descendants. Indigenous people determine their beliefs and keep knowledge and language systems, and it has been noted that identifying rather than defining indigenous people is a more productive means to successful description [63]. Worldwide, it is estimated that 5% of the global population, 370 million persons, are indigenous. Notably, while 65% of land is owned under indigenous right, governments do not recognize the entirety of this land as indigenous [31].

The indigenous peoples of the Americas are diverse. According to the Pan-American Health Organization (PAHO), a WHO collaborative, there are over 400 identified indigenous groups that together comprise about 45 million indigenous people in North, Central and South America [66]. Of these 45 million indigenous persons, 33–40 million live in Latin America (Central and South America) and the Caribbean [66]. In the United States, 566 tribes comprise 1.7% of the total population that identify as partially or fully Native American or Alaskan native (International Work Group for Indigenous Affairs) [33]. Native Canada indigenous are Inuit, First Nation and Metis, and these indigenous comprise 4.3% of the Canadian population [33] Latin America is comprised of many indigenous groups, and prevalence varies dependent on country and statistical diligence.

Incorporating, governing and assisting indigenous groups is a complex issue. Statistics and census information on indigenous are imperfect, as even the term indigenous is often interpreted differently and recognized variably by governments. Acknowledging this, basic understanding has been agreed upon through classification by several criteria, including community attachment and distinct language [30]. Movements to improve indigenous data and policies are seen locally and globally, through efforts of the United Nations (UN), World Bank and peer organizations. These movements seek to increase indigenous voices at policy tables, aggregate accurate indigenous information through inclusion and assure indigenous rights through protections [16, 31, 63, 64].

Additionally, complexities in health and wellbeing of indigenous are prevalent. Healthcare access and delivery is multifaceted for the indigenous. It is estimated that 80% of the population in developing countries practice traditional medicine, though the indigenous often incorporate Western and new medicine into these practices. In fact, traditional medicine continues to be the most widely practiced method of healthcare in developing countries, yet significant measured health disparities exist between indigenous groups and non-indigenous groups. In example, in Latin America, infant mortality among indigenous children is 60% greater than non-indigenous. Too, while the indigenous in Canada represent 4.3% of the total Canadian population, they represent 19% of TB disease burden in the country [62]. Environmental concerns threaten infectious disease increases, reproductive health, food sources and reduction in traditional plant medicine [19, 20, 26, 57]. Socioeconomic disparities threaten poor health outcomes associated to poverty, and prejudice against indigenous has affected access to services [10, 24]. The health of the indigenous continues to be of concern worldwide.

Current efforts to achieve parity for the indigenous, as it is interpreted worldwide, include basic access to education, resources, sustainable ecosystem and health all within acceptable cultural standards. The International Indian Treaty Council (IITC), one of the first non-governmental organizations to work on behalf of the Western hemisphere indigenous, was formed in 1974. The IITC brought together peoples from North, Central and South America as well as the Caribbean and Pacific [32]. Within several decades, multiple organizations became advocates for the indigenous, adopting guidelines and drafting resolution to work toward indigenous equity and voice. The United Nations Indigenous Peoples Partnership was formed in 2011 to work toward the UN resolution on indigenous people, the United Nations Declaration on the Rights of Indigenous People (UNDRIP) passed in 2007. This declaration prohibits discrimination of indigenous people and works toward ensuring rights, culture and ethics are preserved for all countries in agreement [64].

As governments, non-governmental agencies and philanthropic organizations work toward addressing and aligning strategic missions on behalf of indigenous people, understanding of individual cultures and the collective indigenous population remain varied. Detailed, accurate focus remains a priority. It remains a global effort to ensure human rights, assure global governance declarations and improve indigenous health equity. Thus, culturally appropriate indigenous health research and delivery, sensitive to both definition and practice, remain a goal in medicine.

## Current issues

There is significant data to represent major differences in indigenous and non-indigenous health. Measures are associated with major outcomes such as mortality, morbidity and life expectancy. Access to services, education and behavioral research is also available. In Latin America, indigenous make up 8% of the population but 14% of the poor. The indigenous are significantly less likely to complete primary or tertiary education and much more likely to live in poverty than non-indigenous peers [69].

A comprehensive study found that the indigenous have high infant mortality and lower life expectancy than non-indigenous, stratified by country, though differences in rates were noted. A total of 148 data sources were used: 115 government sources, 22 academic sources, 11 sources of other agencies such as UN and WHO [4]. In this calculated study, there was a 4.8 year life expectancy increase for general US populations compared to American Indians and Alaskan NAtives. There was a 5.5 year life expectancy increase for the general Canadian population compared to First Nations population. There was a 17 percentage point difference in Peru between children under 5 years of age with stunting. There was a 20.4 point difference between Colombian indigenous and Colombian general population for infant mortality. Indigenous Chilean children aged 6–7 were less obese than the benchmark population. Brazilian indigenous infants were more likely to be born with low birth weight, In Venezuela, non-indigenous infant mortality was much lower than indigenous infant mortality, a difference of 24.5 points [4].

There is a wide variance on methodology and tools used for qualitative and quantitative data collection, much of it dependent on government recognized indigenous classification. In the United States, CDC reports of health disparities have concluded that colorectal cancer screening prevalence is lowest, binge drinking is highest, motor vehicle crash rate is highest and drug induced death rate are highest in American Indian/Alaskan Natives (AI/AN) compared to other ethnicities in the U.S. Additionally, smoking prevalence is highest in AI/AN youth compared to peers [12]. These reports utilize a wide range of tools, including telephone surveys such as Behavioral Risk Factor Surveillance System (BRFSS), death certificate data used in National Vital Statistics System (NVSS) and the National Survey on Drug Use and Health (NSDUH) [12]. Despite a paucity of accurate and standardized indigenous health disparities, there is an abundance of varying literature on the overall issue.

Besides census and overall public health outcome data, qualitative data has provided insight into repetitive patterns. One recent systematic literature review of 60 publications highlighted significant qualitative data that demonstrates major discrimination of Latin American indigenous in the healthcare setting. This study also highlighted potential short term and long term health outcome effects of the discrimination and major misuse and disuse of the healthcare system by the indigenous as a result of discrimination [10].

Efforts to address indigenous disparities are often tailored to one group or region. Methodologies are inconsistent. Additionally, conclusions can remain vague. In example, a Peruvian effort to decrease disparities between indigenous and general population maternal health utilized United Nations International Children’s Emergency Fund (UNICEF) expertise. Maternal houses that were tailored to indigenous culture, vertical birthing chairs and language requirements were established in healthcare facilities. There are mixed interpretations of success, with some journals noting the program successes while Amnesty International having reported less successes in patient and physician satisfaction [3, 10]. In contrast, a clear successful example of cultural inclusion for the indigenous was a decrease in infant mortality and acute malnutrition of children in Colombian indigenous. This decrease was accomplished through community based education in Wayuu language. This change was a result of education and cultural perception change in the rights of the child, a concept not reported to be similarly perceived from ancestry of this indigenous group. Permanent resource support and ongoing community effort assisted in the measured success [15].

Finally, studies of health outcomes, health equity and health comparisons for indigenous groups struggle to account for perception and perspective. While cultural norms continue to represent differences among indigenous and non-indigenous populations, whether or not these are disparities are often a matter of perspective. In instance, most analyses and reports on young motherhood consider some indigenous communities’ high prevalence of young mothers as a disparity, regardless of the community’s perspective and cultural norm. In socioeconomic perspective, what one society may call poverty another may view as wealth in natural and human resource. Philosophical and ethical approaches in perspective may assist in cultural competence. Indigenous people experience inequalities in their native countries though the interpretation and assessment of these inequalities has only recently intensified.

Current research on indigenous health does not account for indigenous conceptualization of health, and this mismatch only furthers tensions between indigenous communities and Western medicine [37]. To date, this concern continues to be overshadowed by measurable outcomes of life expectancy, morbidity and mortality. As health perception and quality of life can be cultural, it is indeed critical to better assess indigenous health definitions. In an effort to better understand health satisfaction, researchers reviewed Gallup poll responses from Latin American citizens. It was found that correlation between life expectancy and health satisfaction, as well as income to health satisfaction, was low. In fact 85% of Latin Americans were satisfied with their health. In an area with significant health indicator differences between non-indigenous and indigenous, Guatemala, 94% of Guatemalans were found to be satisfied with health [43]. There have been recent research undertakings to better understand and address research differences for indigenous and general population health analyses. One report notes that indigenous populations utilize socio-ecological context for research hypotheses while traditional research attempts to remove variable contexts in spirit of generalizability [61]. Thus, evidence, data and knowledge transfer interventions are all affected by differences in perception, goals and understanding of health. Additionally, as clearer understanding of indigenous health satisfaction and quality are attained, as well any impacts to greater general populations, a general consensus on provisions must be attained.

Diseases of major public health burden are a primary focus in American Indian/Alaskan Native (AI/AN) studies. Cancer is a primary theme in original data and research on this population. It has been noted that AI/AN women with endometrial cancer were compared to U.S. non-hispanic white women, and no differences in outcome of endometrial cancer were observed. Service delivery has also been assessed. In example, equal proportions of AI/AN and Non-hispanic white women received lymphadenectomy services during surgical stages, but AI/AN women received less adjuvant radiation therapy [45]. In another study, breast tissue was examined using biomarkers to address the disparities in breast cancer mortality between Alaskan Native and Southwestern American Indian populations (a three-fold difference) and resulted in no significant findings [38]. Too, a recent study reported that AI/AN men were less likely to report a timely prostate screening as compared to white and black men in America [25]. In cancer screening studies, it was found that AI/AN persons had lower rates of colorectal screenings overall compared with white and black populations, though public health messaging succeeded in AI/AN screening improvements [36].

Studies involving infectious disease also help to describe inequities in AI/AN care. Lower respiratory tract infection hospitalization rates in pediatric AI/AN populations have been higher than for the general U.S. population, though these rates have improved throughout the years [60]. Additionally, it has been noted that the Inuit are at risk for zoonotic infectious disease, particularly with temperature and climate change [23, 29].

Interventions to improve health equity for Pan-American indigenous are specific. Targeted programs have increased colorectal cancer screening for indigenous of the Hopi tribe [8]. A randomized controlled trial found that there was no improvement in cancer screenings among American Indian populations after a calendar mailer campaign [18]. A diabetes prevention program incorporated classroom technique and resulted in sustained weight loss, lower blood pressure and a decrease in diabetes incidence within American Indian populations as well [34]. A motor vehicle accident prevention campaign that targeted American Indian tribes in Wisconsin and Arizona resulted in an increase in child safety seat and seat belt use as well as a decrease in alcohol impaired driving [6].

Attitudes and perceptions continue to be assessed among AI/AN populations. In example, prevention for the human papillomavirus by way of the HPV vaccine was assessed in a focus interview study, and subjects reported that concerns over safety and lack of knowledge contributed to issues surrounding HPV vaccine acceptance within Northern Plains populations [56]. In a survey study, Alaskan Natives acknowledged awareness on dangers of smoking and a willingness to quit, but they were half as successful as non native Alaskan Natives [54]. Alaskan Natives also reported that tradition and culture in their indigenous population is a protective factor against suicide, though Alaskan Native suicide rates are much higher than the general US population [17]. Attitudes and perceptions are important not just for cultural awareness; they are important to describe inequity and healthcare malalignment in even greater detail.

Canadian indigenous research also provides insight into diseases of major public health burden. In instance, compared with the general Canadian population, Aboriginal pediatric cancer patients had lower 5 year survival rates and less 5 year event free reports [46]. Aboriginal youth were also the subjects of a smoking survey that revealed a higher prevalence of Aboriginal youth smokers compared to the general Canadian population. This finding matches indigenous groups in Australia and the United States [21]. First Nation women have a poorer survival rate with breast cancer diagnoses compared to non-First Nation Canadian women. With the exception of diabetes, if comorbidities are present, First Nation women with breast cancer experience a mortality risk 5 times that of non-First Nation women [59]. First Nation adults experience a higher risk of both end-stage renal disease and death without end stage renal disease, though reasons are complex and future research on differential mortality is warranted [35].

Latin American indigenous studies represent numerous communities and countries. Still, studies are focused and detailed, ranging from infectious disease to chronic conditions to maternal and child health. The indigenous populations are often compared to the full population using country borders as a the classification. In a national survey study throughout Brazil, indigenous women were found to have a higher prevalence of obesity, anemia and hypertension. Indigenous children under the age of 5 were found to have higher prevalence of recent diarrhea, recent hospitalizations, anemia and height for age deficits [14]. Xavante village children in Brazil were reported to have a high prevalence of malnourishment, represented through weight for age and height for age deficit that were greater than other documented national surveys [22]. A Guatemalan nutritional health study also examined maternal and child associations, with child stunting associated with Guatemalan indigenous [41]. A Peruvian indigenous study found no difference in provider treatment, technical or socioemotional, among Peruvian indigenous and non-indigenous in health settings [53]. The Mexican Maya-Yucatan indigenous were subjects of two studies, both observing high prevalence of arthritis and musculoskeletal disorders alongside disability [44]. Pan-American indigenous are found disproportionately in agricultural labor camps, exposing them to higher risks of vector borne illnesses. Amazon forest communities have a higher prevalence of parasitic disease [29]. Inequities in Latin American indigenous communities are clear and diverse.

Indigenous disparities are also studied and described without major population comparison. Vitamin D was also assessed in Ecuadorian Andes, where it was found that Vitamin D deficiency was significantly prevalent among the indigenous despite abundant sunlight [49]. In another example, researchers noted the direct sexually transmitted infection rate for Paraguay indigenous women was 41.4%, and a quarter of these women were found to have more than one STI [47]. Too, Brazilian indigenous residing in forests were also assessed in nutritional health. Protein adequacy discrepancies between children and mothers as well as between boys and girls were found. This study quantified food scarcity in observation of maternal protection to food insecurity [52]. Research on a remote Peruvian region comprised of mostly indigenous peoples found an association between biomass fuel use and increased risk of hypertension and higher blood pressure [9]. A current Guatemalan report seeks insight on indigenous perinatal mortality after construction of 68 centers for labor and delivery [39].

Genetic understanding and disease course have been the subject of interest in medicine for quite some time, though few studies involving the indigenous have appeared. A recent study incorporating genetics was completed in Mexico, however, and compared TB disease. There was a higher proportion of indigenous ancestry in those with TB disease than LTBI, a 4.65% greater Native American genetic ancestry in fact [70]. The importance of incorporation of indigenous genetics research is clear.

Another factor weighing into indigenous consideration is changing ecosystems. Environmental degradation and destruction in indigenous areas is an ongoing issue, with deforestation and ecosystem changes affecting food supply, communicable disease control of vector borne illness and cultural norms. Deforestation in the Amazon regions has led to loss of medicinal plant species. It has also led to widespread concern over supply as well as concern over chemical and biological understanding of the traditional medical plant uses [57]. As studies have shown indigenous use forest species more often than non-indigenous, it is critical to maintain vigilance in deforestation and environmental health indigenous impact awareness. Equivalent to removing community pharmacies, actions that affect both environmental health and indigenous health must be studied, regulated and poor outcomes prevented [57]. Too, bioscience pharmaceutical replacements may present future alternatives, yet indigenous health behaviors may not move in tandem. Food supply and subsequent effect on health must also be at the forefront of environmental indigenous health. A recent study of an Inuit settlement region found that changes to the freshwater ecosystem were leading to adaptable but less than ideal changes in food supply, including financial strain and changes to preferences in drinking water access [26]. Strategies may alter but not completely eliminate the effects of external force destruction to indigenous areas. In example, food supply may be modified once agricultural areas are affected, but changes to the indigenous diet leads to new health concerns. Projections in surface temperature increase may increase the spread of zoonotic diseases such as giardia and brucellosis in Northern American indigenous populations that depend on food supply in areas affected [23]. Temperature changes as a result of climate change may alter flood cycles in the Amazon as well as change incidence and prevalence of vector and waterborne diseases such as dengue, yellow fever, malaria and cholera in the Amazon [23]. Destruction of ecosystems also affects disaster planning and prevention [62], a necessity that the indigenous often are not afforded.

In examining the health of the indigenous, it is imperative to also acknowledge delivery analytics. Healthcare delivery in many countries worldwide has grown through universal health coverage (UHC). UHC has become a global goal through WHO and UN initiatives, and UHC seeks to ensure all people can use promotive, preventive, curative, rehabilitative and palliative health services necessary without incurring financial hardship. UHC strives to accomplish this goal by ensuring protection from financial risk, restructuring financing in health services, ensuring quality of services that drive improvement and ensuring health equity. In examining UHC and indigenous, there are several lingering intracices. UHC is not yet a perfected approach worldwide. Efforts to assure access to basic healthcare varies by country, with imperfections accompanying each pathway. Too, UHC is supported by the moral compass that healthcare is a human right. Not all governments support this and not all governments assure equal protections for indigenous [51, 58].

Finally, UHC strives for health equity yet has been noted to be a risk itself in further marginalization of indigenous populations. UHC hinders on community or population agreement of health goals. Indigenous may define health, healthy outcomes and health satisfaction differently. Too, indigenous may view ecosystem, land and other tension-inducing considerations as components to health access. Traditional medicine, water, land and community decision making is not usually under UHC structure [68, 70]. Therefore, there is a need for modeling and best practice approaches in UHC as the health of the indigenous advances.

## Recommendations

Recommendations to improve indigenous health in effort of health equity are variable and feasible. Stronger epidemiology, continued cohesive global strategy and better research alignment with emphasis to quality are all strategics means to health parity for Pan-American indigenous groups. Additionally, comprehensive metrics analysis in healthcare delivery can transform the health of the indigenous through improved conceptualization, analytics and healthcare delivery.

Stronger epidemiology through definition and general data on Pan-American indigenous is vital. Accuracy in census and statistics of indigenous populations are of debate, particularly with regional variables in census collection and analytics [16]. Leading organizations such as the World Bank rely on regional and national census, as well as secondary data, for estimates of indigenous populations [69]. Census concerns for indigenous populations are so dynamic that the United Nations has produced significant work to train, enhance and strategize best practices in data collection and surveillance of indigenous populations. As some governments do not even recognize indigenous populations, best practices in future data aggregation must also include indigenous leadership. Indigenous associations and organizations provide continued voice at the table. Additionally, the UN has recently established a forum within the UN Permanent Forum on Indigenous Issues (UNPFII) that establishes indigenous representatives for increasingly assertive leadership [16]. There has also been a newly established Indigenous Navigator, an online tool and portal dedicated to indigenous data, statistics and information for the purposes of accuracy and advocacy [1]. Identification and accounting of indigenous populations remains subpar, yet strategies continue to seek best paths forward.

Healthcare administration for indigenous populations, such as the Indian Health Service (IHS) and First Nation and Inuit Health Branch, should work to ensure all coded terminology and definitions are matched and standard with national and private payer plans. This assists researchers in accuracy and maximizes data collection. Data collection should also take care to stratify for indigenous, in a manner that assures privacy and minimizes potential for bias in countries still affected by indigenous prejudice, as well. In example, only four countries in Latin America account for indigenous status in cancer registries, despite ongoing and strong genetic associations to the disease [48]. Lack of epidemiological framework has been identified as a concern in the Caribbean region [55], and indigenous communities are of even greater disadvantage from this knowledge gap. Additionally, Native Hawaiian and American Islanders are often grouped in census studies as Pacific Islanders, despite national borders being primary population comparisons. To better understand indigenous at country and governmental level, it may be of future benefit to revise these identification classifications in indigenous research. Efforts to strengthen collective data on indigenous communities can be individualized by country and region and assisted in overall structure with global guidance and agreement.

Continued efforts by global governance for global indigenous and Pan-American indigenous health equity should be encouraged. Collaborations that bring indigenous leadership voice to the table and drive indigenous data accuracy must be funded. Additionally, these efforts must be continually followed up on, with stakeholder reports, accountability and timelines all delineated. Needs assessments in Pan-American indigenous parity programming should be detailed and matched to sustained funding. Finally, research and interventions must be strategic, built from previous work and transparently accounted for. Rather than piecemeal work unknown outside of a university program, research and interventions should be searchable, known and discussed in real time.

Funding and research priorities must be aligned. While the majority of Pan-American indigenous live in Latin America, research on these communities lacks scope and priority. Funding and collaborations should take care to prioritize all indigenous communities in research representation. National and global health agencies should strive to encourage more research for Latin American and Caribbean indigenous and seek to maximize current research projects. Additionally, priorities of general population and researcher knowledge must be balanced with priorities among indigenous, with consensus established.

There are clear strengths and limitations in indigenous research that must be addressed. Small sample size is expected for population studies of small communities. The diversity of indigenous groups make research findings less generalizable. However, recent literature has highlighted potential solutions to sampling concerns, including combination sampling, community organization assistance and comparison strategies [7].

Another consideration to maximize research for the indigenous lies in data collection methods. The most commonly used methods of data collection include health record review and household surveys and interviews. Data collection methodology and health terminology varies, from healthcare organization to region to country. Codes and definitions within healthcare organizations, private payers and government administrations may at times vary, hindering accuracy in research. Household surveys and interviews rely on clear communication, and language barriers between indigenous and non-indigenous exist. Translator services and indigenous leaders may assist, but data collection between researchers and families cannot be accurately representative with communication disconnect. Additionally, indigenous self-reporting to researchers risks recall bias, a significant limitation. Along this same path, qualitative data impact should be better understood. Focus groups provide qualifying data, but the true impact of this data has yet to be identified within the literature. Research should take care to minimize potential limitations, particularly as research studies on indigenous are not prevalent or well funded.

Cancer is a primary health issue worldwide, including for indigenous populations. Data continues on cancer and indigenous. In example, a recent study found no link between prostate-specific antigen (PSA) levels and age in a tribe of the Brazilian Amazonian forest region. This data provides better insight into biology, genetics and guidelines for screening [42]. Yet not only is better data collection and epidemiology methodology necessary for cancer and indigenous health equity, full understanding of interventions and healthcare operations to prevention and management of cancer is critical. In example, recent data on Inuit provided ability to establish risks regarding cancer. While prostate cancer remains a low risk to Inuit, nasopharyngeal cancer is an extremely high risk to the Inuit. Too, this study noted that Inuit have the world’s highest incidence of lung cancer [71]. Based on studies of surveillance epidemiology, future research and health equity policy can revolve around early detection, prevention and education and vaccination.

Additionally, it is important to understand indigenous behaviors and knowledge around cancer. Cancer incidence has risen in First Nations people of Canada, and additional research has noted that a lack of culturally appropriate education and expertise, inadequate access to services, poor understanding of prevention and treatment and a mistrust in the current health system all contribute to poor cancer journeys for the Canadian indigenous. Subsequent planning has led to a strategic government partnership agenda to address inequities and disparities [5]. Additionally, research has indicated suboptimal knowledge, attitudes and beliefs on cancer screening among indigenous communities. Greater understanding of barriers and interventions for cancer screening among indigenous communities has been recommended [40]. Additionally, cultural differences in indigenous point to necessary research in palliative care, hospice and survivorship components to cancer. A recent survivorship study recommended tailored, specific approaches to indigenous cancer survivors based on needs, preferences and barriers. Research within specific indigenous and Pan-American groups could advance health equity in cancer survivorship, palliative and hospice care even further [11]. Cancer is multifaceted, with continued unknowns in genetics and environmental correlations, and delivery of care is just as complex. Opportunities in research are noted due to a spotlight on indigenous health focus on cancer.

Shifts to chronic disease focus should not negate acute and infectious disease responsibilities in research. HIV/AIDS and tuberculosis continue to be of concern for the indigenous, particularly given barriers to socioeconomic resources, barriers to healthcare access and barriers to health education. Research on HIV/AIDS and the indigenous has led to several interventions, including language barrier removals [27]. Parasitic infections are of significant concern to Amazon forest communities in Brazil and Venezuela, and a recent study noted less than expected eye damage in the population with afflicted an eye parasite [28]. A recent study on high tuberculosis prevalence among Paraguay Chaco indigenous, eight times that of the general population, noted that poor access to services was a significant association to active tuberculosis disease in children and adults [65]. Additionally, Human T-cell lymphotropic virus type 1 (HTLV-1) and HTLV-2 greatly affect indigenous populations of Latin America [2, 50]. These viruses must continue to be under surveillance, evaluation and research.

It is not only infectious disease epidemiology, etiology and access to care that must be studied. Socioeconomic conditions contribute to infectious disease. Successful interventions that acknowledge socioeconomic conditions can drastically improve quality of life for the indigenous. In example, within the Argentine region of the Gran Chaco ecoregion, which also includes sections of Bolivia and Paraguay, early household notification surveillance and community widespread spraying treatment led to a major decrease in triatomine, the major vector for Chagas disease. This research could be expanded to the Bolivia and Paraguay territories as well as expand in human screening and treatment of Chagas to correlate to triatomine elimination [24]. Studies such as these provide foundations for continual data analytics. Compounding data and literature reviews should be standard health activities as countries look to tackle infectious disease and acute conditions that affect indigenous health equity. Associations, stronger surveillance and socio-ecological factors must be better analyzed on an ongoing basis for infectious disease. It is important to understand inequities in care, not just for one disease but to provide an accurate overall picture. Impactful, targeted data is critical and provides continued focus on acute and infectious disease.

Importantly, the research methods and movement from academia represent the start of a firm foundation and opportunities lie in research quality improvements. Several recent studies have revealed that data collected was the first collection on behalf of the population. This was true despite the fact that the data was collected for basic census and public health agenda, such as mortality, nutrition and child development. Healthcare systems can be designed with sustained research, automatic and fluid in construction. Publication and research quality diligence should ensure that literature review and previous data is easily accessible and acknowledged in matched research. Integrity in research on the indigenous has been reviewed, too, and it was noted that basic research components may not be optimal. Community informed consent is not always clearly obtained, indigenous access to studies and results is suboptimal, indigenous authorship is rare and applicability of research is not always clear. Additionally, community engagement studies are extremely variable in geography, time and date [37]. Researchers should take great care to heed these concerns and apply planning for quality research in study design.

While biological and socioeconomic understanding is the focal point of much of the indigenous research, health service delivery research opportunities with these studies are clear. Healthcare delivery data should be studied in conjunction with all indigenous research as best possible. Native American, Alaskan Native and Canadian aboriginal research is established in conjunction with healthcare administration oversight and participation. As Latin American and Caribbean countries initiate and grow health infrastructure, administrations tasked with indigenous focus could look to these agencies for foundational design. Additionally, global agencies can assist in direction of indigenous research in effort for equitable representation. Underrepresented populations in research, such as the Caribbean indigenous, should be noted as a future priority. A clinician in search of Caribbean indigenous health research should not do without, and all agencies responsible for national and international health should acknowledge disparity and strive to create parity in indigenous research. Prioritization efforts for the indigenous not only aid health and science, they set the tone for social equality. Achieving true social equality for indigenous populations is a constant effort. Research can be maximized in efficiency, cost and impact value through initiating best practices and scaling them.

There have been several noted models in the literature that are successful in research structure. Active and honest relationships that included Native American leadership in research provided successful outcomes to a collaboration between academia and Native American communities. This collaboration also resulted in a position specific for assistance of research facilitation within the Native American community [13]. Literature reviews and meta-analyses can provide deep insight and direct recommendations that nations and communities can move forward on, without hesitation. In another example, Canadian indigenous health disparities were reviewed alongside social determinants of health and socioeconomic conditions affecting disparities, and direct recommendations offered for immediate implementation [40]. Analyses such as these provide direction and should be followed.

Inclusion of indigenous priorities and leadership is a must. There is a notable difference in inclusion of North American indigenous in literature more than Latin American indigenous in literature. If healthcare is to approach indigenous communities, trust and sustained relationships are critical. Researchers should strive, regardless of academic partnership, to establish sound health access and sustained provisions when initiating studies involving the indigenous. In example, if household surveys and data measure child wellness and health, continued and ongoing pediatric services should then be available to these communities. Should blood pressure be measured over several months during a study, regional primary care should then be established and accessible to these communities. Piecemeal research is a missed opportunity for healthcare delivery to the indigenous, a missed opportunity for ongoing data collection and a fractured relationship that must be reintroduced with another project. Barriers to underrepresentation in research have been found to be the same as barriers to healthcare delivery and include distrust, financial barriers, employment and communication as well as literacy gaps [7]. Therefore, solutions to increase research representation and equity may translate to healthcare delivery representation. Established and sustained healthcare should be part of the equation in indigenous research.

The very concept of research hypotheses and methods alter with indigenous perspective. Indeed, researchers have begun to approach concerns of indigenous and health leadership on differences of definitions of knowledge, evidenced based medicine and similar health concepts. One approach described with optimism is that of realist review. In a realist review, researchers can test theory by seeking to uncover key concepts in program mechanisms and contextual factors that contribute to success or failure. This approach allows for an opportunity to bridge traditional Western medicine research methodology with indigenous socio-ecological context [61].

As globalization changes economic and healthcare landscape worldwide, Pan-American countries must be prepared to deal with changing health priorities. Nutritional intake, pharmaceutical access and health regulations strengthened through stronger government participation will affect indigenous communities. A shift from addressing acute injury and infectious disease to addressing chronic conditions can be more successful with country preparation. Too, healthcare demand is expected to grow. Movement from scarcity to healthcare access and better understanding of genetic predispositions are two reasons for an expected increase in healthcare demand, and healthcare supply must be available. A pivot from piecemeal charitable healthcare delivery in remote regions to structured delivery is achievable, with collaboration, consistency in approach and assistance in development. Global agencies should assist in planning, implementation and ongoing evaluation for these nations, and Pan-American jurisdictions must follow suit.

Consideration of health coverage, access demands, UHC and proposals of best practices to Pan-American indigenous inclusion must be critically reviewed. A recent journal highlighted a commons healthcare system, driven by shared community resources of a Malaysian indigenous group. It was determined that natural resource base, knowledge base and social protection are features of a commons healthcare system in which community ownership achieves optimal health [67, 68]. Yet while this approach may be deemed successful in one region, applicability to other regions should be weighed. Should Pan-American countries seek best options forward for indigenous health equity within UHC systems, modeling examined proposals must not be done without thorough examination of supporting evidence. Additionally, as many Latin American countries and Canada have had established government systems for health coverage, it would be of great benefit to have annual reports that include all UHC components for comparison. A recent presentation noted longevity of coverage, as well as definition of population covered, for Latin America. Consideration of the indigenous was a concern. Policy convergence, policy implication and planning by way of anticipation were all strategies for UHC buildout [58], strategies reiterated in many Pan-American global governance reports [51]. These strategies should be continually assessed for indigenous inclusion, with indigenous leadership at the universal coverage table.

Comprehensive measures of health and value are critical to address and retain. As global agencies, non-governmental organizations and governments look to shape strategies to assist in indigenous wellbeing, value and perception must be accounted for. This can be modeled in Pan-American indigenous accountability. Poverty, health and material wealth values, as well as how global definitions of poor accurately describe communities, must be better understood. Indigenous perceptions of these values, from wealth in material to wealth in people and community, should be measured and clear. Western medicine may not be appropriate, may not provide happiness and may not provide peace to all communities. Provider training, service delivery, treatment availability and coverage must account for cultural differences and strive for best response to the community. Measures that indigenous deem as critical to health, measures that include universal coverage scope, measures of health satisfaction and measures that speak to quality of life must all be comprehensive for Pan-American indigenous groups. These measures and reports must be a shared responsibility of the Pan-American populations and governments.

Finally, it is an enormous undertaking to retrieve and review all current published literature on all indigenous populations in the Western hemisphere, particularly due to the nature of publications, narrowly focused studies, disproportionate research on the North American indigenous and the terminology surrounding the populations. While this review sought to summarize current overall indigenous health concerns and recommendations, utilizing global governance reports, Pan-American focus and examples of individual studies, the review was not exhaustive. Future commentaries and all formal literature reviews should strive for comprehensive inclusion of all pertinent research. Methodology, tools and inclusion of socio-ecological and health satisfaction measures should be analyzed and reported on. Additionally, countries’ demographics and inclusion of indigenous in country metrics must be accounted for if data is relied upon. It has been suggested that research and publication quality should strive for major literature review acknowledgement in effort for studies to build with one another. Research, interventions and progress for indigenous health equity will benefit greatly from stronger research and publication quality requirements.

## Conclusion

Current health disparities continue to present between Pan-American indigenous populations and general populations. These health disparities are not yet fully understood due to suboptimal data and analytics. There are significant global and indigenous advocacy groups that have advanced strategic agenda toward indigenous equity for several social issues, including healthcare. Academia, clinical organizations and government institutions have laid the foundation of research collaboration within some indigenous communities, however research must drastically improve. Analyses of indigenous groups comes with significant critique of both epidemiological delays as well as poor research and intervention quality. Recommendations to improve indigenous health in effort of health equity are variable and feasible. Stronger epidemiology, continued cohesive Pan-American and global strategies, better research alignment with emphasis to quality and comprehensive metric analyses in healthcare delivery are all avenues to improve the health of the indigenous. Research and healthcare delivery on the Pan-American indigenous must be maximized for optimal results, must be representative of the indigenous communities, must be implemented in best practice and must introduce sustainable healthcare delivery for Pan-American indigenous health equity. It is critical that healthcare research and delivery be maximized, consistent in approach and collaborative.

With solid organization foundations already established and recommended strategies for research and healthcare delivery optimization, the Pan-American indigenous can experience improved health equity. Additionally, models for worldwide indigenous health equity can be initiated from strong Pan American indigenous health foundation. From these roots, pathways to global harmony for indigenous communities will be continued, paced and successful.

## Compliance with ethics

This article does not contain any studies with human participants or animals performed by any of the authors. The author has no conflict of interest or funding sponsors to declare.
